# SIRT or TACE in combination with lenvatinib and PD-(L)1 inhibitor in high-tumor burden hepatocellular carcinoma: a multicenter, retrospective, propensity score-matched study

**DOI:** 10.1097/JS9.0000000000004543

**Published:** 2025-12-16

**Authors:** Jingjun Huang, Huimin Niu, Hui Zhang, Mingyue Cai, Zhiheng Wang, Yongjian Guo, Licong Liang, Liteng Lin, Tingqi Yang, Jiabai Huang, Yuchan Liang, Zhaoxiong Guo, Jingwen Zhou, Wensou Huang, Kangshun Zhu

**Affiliations:** a Department of Minimally Invasive Interventional Radiology, The Second Affiliated Hospital of Guangzhou Medical University, Guangzhou, China; b Guangdong Provincial Engineering Technology Research Center for Interventional Oncology and Precision Drug Delivery, Guangzhou, China; c Department of Minimally Invasive Interventional Oncology, Jinshazhou Hospital of Guangzhou University of Chinese Medicine, Guangzhou, China; d Department of Hepatobiliary, The First Affiliated Hospital of Army Medical University, Chongqing, China; e Department of Interventional Radiology, Hainan Cancer Hospital, Haikou, China

**Keywords:** cohort study, hepatocellular carcinoma, immunotherapy, lenvatinib, selective internal radiation therapy, transarterial chemoembolization

## Abstract

**Background:**

The combination of transarterial chemoembolization (TACE), lenvatinib, and PD-(L)1 inhibitors (TACE-L-P) has shown improved outcomes in hepatocellular carcinoma (HCC). This study aims to compare the efficacy and safety of selective internal radiation therapy (SIRT) plus lenvatinib and PD-(L)1 inhibitors (SIRT-L-P) versus TACE-L-P in Chinese HCC patients with high-tumor burden.

**Materials and Methods:**

This retrospective, multicenter, propensity score-matched study included HCC patients, beyond up-to-seven criteria or with portal vein tumor thrombus, treated with SIRT-L-P or TACE-L-P across four Chinese centers (2022–2024). Study endpoints included progression-free survival (PFS), overall survival (OS), objective response rate (ORR), and safety.

**Results:**

A total of 234 patients were included (109 in SIRT-L-P, 125 in TACE-L-P). After matching, 91 patients per group remained with well-balanced baseline characteristics. SIRT-L-P demonstrated significantly improved median PFS (12.1 vs. 9.0 months; HR 0.647, *P* = 0.021), OS (2-year OS rate 68.9% vs. 39.1%; HR 0.517, *P* = 0.014), and ORR (78.0% vs. 61.5% by mRECIST, *P* = 0.017; 56.0% vs. 35.2% by RECIST 1.1, *P* = 0.005) compared to TACE-L-P. Safety outcomes showed a lower overall incidence of adverse events (AEs) in the SIRT-L-P group compared to TACE-L-P (any grade: 83.5% vs. 95.6%, *P* = 0.008), while grade 3 AE rates were not significantly different (24.2% vs. 30.8%, *P* = 0.319). In a subset of patients receiving SIRT-L-P, risk tier combining post-SIRT neutrophil–lymphocyte ratio and peripheral CD8+ T-cell proportion was an independent predictor for PFS and OS.

**Conclusion:**

This study provides the first real-world evidence indicating that SIRT-L-P significantly improves efficacy and safety compared to TACE-L-P in high-tumor burden HCC.

## Introduction

Hepatocellular carcinoma (HCC) remains a global health challenge, with over 80% of patients diagnosed at intermediate-advanced stages where curative therapies are often unfeasible^[1]^. Transarterial chemoembolization (TACE) is the standard of care for intermediate-stage disease and has also been widely explored in select advanced-stage cases^[2]^. However, its efficacy diminishes in high-tumor burden scenarios, particularly in HCC exceeding up-to-seven criteria or with portal vein tumor thrombus (PVTT), where median overall survival (OS) was only 10–15.9 months and a median progression-free survival (PFS) approximately 4.8–7 months^[1–3]^.

The integration of molecular targeted agent (e.g. lenvatinib), and PD-(L)1 inhibitors has emerged as a potent strategy to counteract post-TACE angiogenesis and immunosuppression^[4,5]^. Landmark trials LEAP-012 and EMERALD-1 demonstrated a median PFS of 14.6–15.0 months for TACE-based triple combinations (such as TACE with lenvatinib and PD-1 inhibitor [TACE-L-P])^[6,7]^, which were significantly better than single or dual component therapy. With the emerging clinical evidences, such triple combination strategies hold transformative potential to reshape the treatment landscape of HCC^[8]^. Yet, these trials predominantly enrolled early-intermediate stage patients with lower tumor burden, while real-world data reveal less optimal outcomes in advanced cohorts^[4,5]^, highlighting the need for more effective approaches.

Selective internal radiation therapy (SIRT), a transarterial locoregional therapy utilizing yttrium-90-loaded microspheres to deliver targeted radiation, offers distinct biological advantages over transarterial chemoembolization (TACE)^[9]^. Unlike TACE’s embolization-driven ischemic effects, SIRT achieves deeper tumor penetration through radiation-dominant mechanisms while preserving nontarget tissues^[9]^, and induces immunogenic cell death via mitochondrial DNA release^[10]^. Clinically, SIRT demonstrates superior control of index lesions in HCC patients, particularly in those with PVTT^[11]^, with reduced abdominal pain and hepatic toxicity compared to TACE^[12]^. Mechanistically, SIRT activates systemic anti-tumor immunity by enhancing CD8 + T-cell and NK cell populations in both tumor microenvironment and circulation^[10,13]^, in contrast to TACE’s immunosuppressive profile characterized by reduced CD8 + T-cell infiltration^[14]^ and increased TREM2 + tumor-associated macrophages^[15]^. Besides, the radiation sensitivity of tumor cells appears to be enhanced by lenvatinib^[16]^, likely due to the vascular normalization effect and the alleviation of tumor hypoxia resulting from the dual inhibition of the VEGFR and FGFR pathways^[17,18]^. These advantages make SIRT a more attractive candidate than TACE for combination with lenvatinib and PD-(L)1 inhibitors in high-tumor burden HCC. However, no clinical studies have reported SIRT-based triple therapies. Meanwhile, the applicability of SIRT to Chinese HCC patients, who often present with distinct etiological profiles (e.g., HBV dominance) and high-tumor burden, remains unexplored in peer-reviewed journals.

Herein, this multicenter, propensity score-matched cohort study aims to address this critical evidence gap by investigating whether SIRT combined with lenvatinib and PD-(L)1 inhibitors (SIRT-L-P) provides superior efficacy and safety versus the TACE-based combination (TACE-L-P) in Chinese patients with high-tumor burden HCC, and preliminarily explore clinical biomarkers to assess post-SIRT immune modulation and early identify patients who could potentially benefit from SIRT-L-P.

## Materials and methods

### Study design and patient selection

This multicenter retrospective cohort study was approved by the local institutional review board (approval number: LYZX-2025-039-01), and written informed consent was waived for its retrospective nature. The study was conducted in accordance with the Declaration of Helsinki. This cohort study has been reported in line with the STROCSS guidelines^[19]^ (Supplemental Digital Content 1, available at:http://links.lww.com/JS9/G422). Medical records were collected and analyzed retrospectively. Patients with HCC who received either SIRT with Lenvatinib plus PD-(L)1 inhibitors (SIRT-L-P group) or TACE with Lenvatinib plus PD-(L)1 inhibitors (TACE-L-P group) at four hospitals in China (hospital names removed for double-blind peer review) from June 2022 to October 2024 were screened. The criteria of combination timeframe were defined as administration of lenvatinib plus PD-(L)1 inhibitor initiated within 1 month before or after the first on-study SIRT (or TACE) procedure and at least one cycle of lenvatinib plus PD-(L)1 inhibitor has been received after the procedure.

**HIGHLIGHTS**

- Selective internal radiation therapy (SIRT) in combination with lenvatinib and PD-(L)1 inhibitors (SIRT-L-P) significantly improved the progression-free survival (PFS), overall survival (OS), and objective response rate in Chinese hepatocellular carcinoma (HCC) patients with high-tumor burden, compared to transarterial chemoembolization (TACE) plus lenvatinib, and PD-(L)1 inhibitors (TACE-L-P).
- A risk tier that combines the post-SIRT neutrophil–lymphocyte ratio and peripheral CD8 + T-cell proportion was an independent predictor for PFS and OS in patients who received SIRT-L-P.
- The combination of SIRT, lenvatinib, and PD-(L)1 inhibitors was tolerable and more effective, suggesting that this triple combination might represent a better treatment option for patients with HCC beyond up-to-seven criteria or with portal vein tumor thrombus.

The inclusion criteria were as follows: (a) age between 18 and 75 years; (b) histologically or clinically confirmed diagnosis of HCC at BCLC stage B or C (extrahepatic metastases were allowed); (c) Eastern Cooperative Oncology Group (ECOG) performance status score 0 or 1; (d) Child–Pugh class A or B7; and (e) unresectable HCC with intrahepatic tumor beyond up-to-seven criteria and/or with PVTT. Up-to-seven criteria were assessed using baseline contrast-enhanced CT or MRI imaging within 4 weeks prior to SIRT (or TACE), with tumor burden score calculated as (number of lesions) + (largest lesion diameter in cm). Patients scoring >7 were classified as beyond up-to-seven. Patients were excluded from this study if there was (a) receipt of other loco-regional therapies, including hepatic arterial infusion chemotherapy, external radiation therapy, or radioactive seed implantation; (b) prior systemic therapy (e.g., tyrosine kinase inhibitors, immune checkpoint inhibitors, or conventional chemotherapy) other than lenvatinib plus PD-(L)1 inhibitor initiated within the predefined combination timeframe; (c) significant arteriovenous shunting that cannot be corrected by embolization during the first on-study pre-SIRT mapping or TACE procedure, thereby precluding safe and effective intra-arterial therapy; (d) history of other malignancies; and (e) incomplete medical records.

### SIRT procedure

SIRT was performed according to standard practices, which included a pretreatment mapping angiogram with technetium-99 m macro-aggregated albumin (^99m^Tc MAA) scintigraphy, for the hepatic vascular anatomy assessment, confirmation of complete tumor coverage, microsphere biodistribution simulation, lung shunt fraction evaluation, and yttrium-90 (^90^Y) dose/activity determination followed by ^90^Y microsphere infusion. During the mapping angiogram, selective hepatic angiography and cone-beam CT were performed to determine tumor arterial supply and encompass all injection sites to establish the perfused volume. Arteries that were angiographically found to be perfusing the targeted tumors were selected by the interventional radiologist prior to the injection of Tc-99m MAA. Prophylactic coil embolization of vessels deemed to be significant in directing microspheres into extrahepatic sites, e.g. the gastroduodenal artery and gastric artery, was performed as necessary. Then 2.5–5 mCi (4–8 mL) of 99mTc-MAA was injected into the selected arteries perfusing the targeted tumors, and a single-photon emission computed tomography (SPECT)/CT imaging within 1 hour after the infusion of 99mTc MAA was performed, which were used to calculate the lung shunt fraction, identify any extrahepatic distribution, assess the intrahepatic distribution of radiation particles and assess the tumor-to-normal liver (T:N) ratio for ^90^Y activity/dose calculation. Patients were excluded from SIRT treatment if lung shunting was >20 %; the radiation dose to the lungs exceeded 25 Gy for a single infusion; the total cumulated dose to the lungs exceeded 50 Gy; the T:N ratio was poor; or significant extrahepatic distribution of the microspheres could not be avoided by super-selective catheterization or prophylactic embolization.

For patients who could proceed to SIRT, SIRT was performed using ^90^Y-resin microspheres (SIR-Spheres^®^, Sirtex Medical) 1–2 weeks after mapping angiogram. A partition model was used to calculate the ^90^Y activity needed for a planned dose ≥120 Gy to the targeted tumor (a minimum dose of ≥100 Gy would be acceptable when safety considerations necessitated dose reduction), while ensuring the dose to the whole nontumoral liver volume remained below 40 Gy and lung exposure below 25 Gy^[20]^. The calculated activity of ^90^Y-resin microspheres was administered in the exact same arterial location as where the Tc-99m MAA delivery occurred. For patients with bilobar disease, if selective treatments were feasible, bilobar tumors were treated in a single session of Y90-radioembolization. At least 30% of the total functional liver volume was planned to be spared from radiation. Sequential treatment was performed at 1- to 2-month intervals for patients with bilobar disease for whom selective treatments were not feasible. All patients underwent positron emission tomography computed tomography of the abdomen within 48 hours to verify the distribution of ^90^Y microspheres.

### TACE procedure

The patients received either conventional TACE (cTACE) or drug-eluting bead TACE (DEB-TACE) according to the recommendation of the treating interventional radiologist and the patients’ choice. For cTACE, an emulsion of 5–20 mL Lipiodol (Guerbet, Paris, France) mixed with 20–60 mg pirarubicin (Hisun Pfizer Pharmaceuticals, Fuyang, China) was administered into the tumor-feeding vessels, followed by embolization with polyvinyl alcohol particles (90–500 μm; Cook, Bloomington, Indiana, USA). For DEB-TACE, CalliSpheres (Hengrui Medical, Suzhou, China) or DC Bead (Biocompatibles, Farnham, Surrey, UK) with 100–300 μm in diameter was used as the drug carrier and embolization agent. Typically, one vial of the beads was loaded with 60 mg pirarubicin. If blushed tumors were still visible after the embolization with one vial of beads, regular microspheres (8spheres, Hengrui Medical, Suzhou, China; Embosphere, Biosphere Medical, Roissy en France, France) with diameters of 100–700 μm were additionally injected.

During TACE, superselective catheterization was performed, and the embolization end point was blood stasis of the tumor-feeding arteries. In patients with huge or bilobar multiple lesions, in order to reduce the risk of complications, the embolization end point was not achieved in the initial TACE but in the second or third TACE session as described in previous study^[21]^. In the case of arterioportal or arteriovenous fistula, the fistula would be embolized with 300–710 μm polyvinyl alcohol particles before administration of the drug-oil emulsion or drug-loaded beads as determined by the treating interventional radiologist. TACE was repeated “on demand” upon the demonstration of viable tumor by follow-up CT or MRI in patients without deteriorated performance status or organ function.

### Lenvatinib and PD-(L)1 inhibitor administration

Lenvatinib (Lenvima^®^, Eisai, Tokyo, Japan) at a dose of 12 mg (bodyweight ≥60 kg) or 8 mg (bodyweight <60 kg) was orally administered once a day. The PD-1 inhibitors sintilimab (Tyvyt^®^, Innovent Biologics, Suzhou, China) and tislelizumab (BaiZeAn^®^, BeiGene, Shanghai, China) were administered at a dose of 200 mg via intravenous infusion once every 3 weeks. Other PD-(L)1 inhibitors including pembrolizumab (Keytruda^®^, Merck Sharp & Dohme, Kenilworth, NJ, USA), camrelizumab (AiRuiKa^®^, Jiangsu Hengrui Pharma, Lianyungang, China), durvalumab (Imfinzi^®^, AstraZeneca, Cambridge, UK), and atezolizumab (Tecentriq^®^, Roche, Basel, Switzerland). The dose reduction (no dose reduction for PD-[L]1 inhibitors), interruption, and discontinuation were conducted depended on the presence and severity of toxicities according to the local labels.

### Follow-up

Patients were followed up at approximately 4- to 8-week intervals after the initial treatment. Each follow-up session routinely included detailed recording of medical history, physical examination, laboratory tests, an abdominal contrast-enhanced CT or MRI examination, and a chest CT plain scan. Laboratory tests included hematologic and biochemical analyses, such as complete blood cell count, prothrombin time, measurement of α-fetoprotein, aspartate aminotransferase, alanine aminotransferase, total bilirubin, serum albumin, alkaline phosphatase, creatinine, thyroid function tests, and urinalysis. Based on prior evidence of post-SIRT immunomodulation^[10,13]^, in a subset of patients who was scheduled for PD-(L)1 inhibitor therapy after SIRT, lymphocyte subset analysis at circulation including peripheral CD8 + T-cell proportion were performed via flow cytometry at baseline and 3–4 weeks after SIRT and before PD-(L)1 inhibitor initiation to observe the immune status of patients.

### Assessments and outcomes

Tumor response was classified as a complete response (CR), partial response (PR), stable disease (SD), or progressive disease (PD) assessed based on contrast-enhanced CT or MRI imaging using the modified Response Evaluation Criteria in Solid Tumors (mRECIST) criteria and RECIST 1.1, respectively. The objective response rate (ORR) was defined as the proportion of patients who were classified as CR or PR. All treatment responses were assessed independently by two diagnostic radiologists with 15 and 12 years of experience in abdominal imaging, respectively. In case of ambiguity in the response assessment, the final determination was made by consensus or a third diagnostic radiologists with 19 years of experience.

Furthermore, we analyzed the progression-free survival (PFS) and overall survival (OS) of the patients. PFS was defined as the time from the first SIRT or TACE procedure until the date that progressive disease according to mRECIST or death was confirmed. In the PFS analysis, patients who underwent tumor resection or liver transplantation prior to documented progression were censored at the date of surgery to avoid overestimating the direct biological efficacy of the initial treatment. In sensitivity analyses of PFS, PFS was re-evaluated without censoring patients at the time of surgery, to examine the robustness of the primary analysis. Under this approach, patients undergoing resection remained at risk until documented disease progression or death. OS was defined as the time from the first SIRT or TACE until the date of all-cause mortality regardless of resection status, as surgical intervention could represent a downstream benefit derived from tumor response of initial treatment.

Adverse events related to SIRT or TACE that occurred within 3 months after treatment were recorded. Adverse events related to lenvatinib and PD-(L)1 Inhibitor were collected until 3 months after treatment discontinuation or until the initiation of new systemic anticancer therapy, whichever occurs first. All adverse events were evaluated according to National Cancer Institute Common Terminology Criteria for Adverse Events version 5.0.

### Statistical analyses

A 1:1 PSM was performed using the nearest-neighbor method without replacement to minimize selection bias. A caliper width of 0.02 on the propensity score scale was applied to ensure proximity of matched pairs. The propensity score (the probability of receiving SIRT-L-P) was calculated by a logistic regression model including variables of age (years), sex (male vs. female), hepatitis B surface antigen (positive vs. negative), ECOG PS (1 vs. 0), Child–Pugh class (B7 vs. A), α-fetoprotein level (>400 ng/mL vs. ≤ 400 ng/mL), tumor size (cm), intrahepatic tumor number (>3 vs. ≤ 3), PVTT (present vs. absent), extrahepatic spread (present vs. absent), prior TACE (yes vs. no) and PD-(L)1 inhibitor (tislelizumab, others vs. sintilimab). Covariate balance was assessed via standardized mean difference (SMD), with |SMD| < 0.1 indicating adequate balance. Sensitivity analyses were conducted to evaluate the robustness of the PSM analysis and primary findings through three different approaches: (1) PSM with a caliper width of 0.05, (2) PSM employing the optimal matching method to minimize global distance, and (3) inverse probability of treatment weighting (IPTW) (detailed in Supplementary Materials). Additionally, to address potential bias from prior TACE, a sensitivity analysis restricted to patients without prior TACE was conducted, in which a 1:1 nearest-neighbor PSM with a caliper width of 0.02 was reperformed.

Categorical data were summarized as number of patients (percentage), and quantitative data were summarized as median value (interquartile range). Chi-squared test or Fisher exact test for categorical variables and Mann–Whitney *U* for continuous variables were applied to investigate the inter-group differences. PFS and OS were assessed using the Kaplan–Meier method, and the log-rank test was used for comparisons between two groups. Univariate and multivariate analyses of prognostic factors with hazard ratio (HR) and its 95% confidence interval (95% CI) for PFS and OS were performed using Cox proportional hazard regression models. Odds ratio (OR) with 95% CI for ORR was calculated using logistic regression model. Overall incidences of AE in any grade and in grade 3 were compared between treatment groups, while specific AE category comparisons were omitted to avoid inflation of type I error due to multiple testing. All statistical tests were two-sided, and *P* < 0.05 was considered statistically significant. All statistical analyses were performed using R (version 4.4.2; The R Foundation for Statistical Computing, https://www.r-project.org/; RRID: SCR_001905) and SPSS Statistics (version 26.0; IBM, Armonk, NY; RRID: SCR_016479).

## Results

### Cohort characteristics

**Figure 1.**
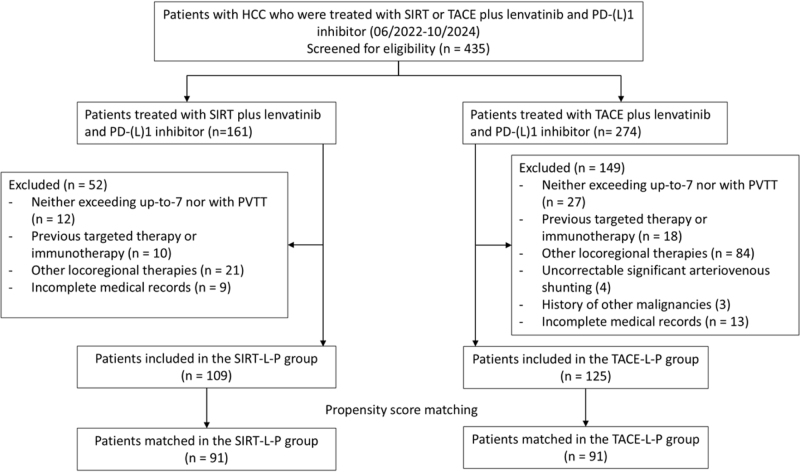
Flowchart of patient inclusion, exclusion and matching in the study. HCC, hepatocellular carcinoma; SIRT, selective internal radiation therapy; TACE, transarterial chemoembolization; PD-(L)1, programmed cell death–(ligand)1; HAIC, hepatic arterial infusion chemotherapy; SIRT-L-P, selective internal radiation therapy combined with lenvatinib and PD-(L)1 inhibitor; TACE-L-P, transarterial chemoembolization combined with lenvatinib and PD-(L)1 inhibitor; PVTT, portal vein tumor thrombus.

A total of 234 patients were included in this study, 109 of whom received SIRT-L-P and 125 received TACE-L-P (shown in Figure 1). Before PSM, |SMD| was > 0.1 for several covariates, which indicated an unbalance between the groups. After matching, 182 patients (91 patients in each group) remained in the study cohorts. The |SMD| was <0.1 for all covariates after matching (|SMD| < 0.001 for several key covariates, i.e. HBsAg, Child–Pugh class, BCLC stage, and α-Fetoprotein level), which indicated well-balanced baseline characteristics between the two matched groups (shown in Table 1; Supplemental Digital Content Figure S1, available at:http://links.lww.com/JS9/G421).

**Table 1:** Baseline patient characteristics before and after matching.

Characteristics	Before PSM				After PSM			
	SIRT-L-P (*N* = 109)	TACE-L-P (*N* = 125)	|SMD|	*P-*value^*^	SIRT-L-P (*N* = 91)	TACE-L-P (*N* = 91)	|SMD|	*P-*value^*^
Age (years)	55 (46–63)	55 (48–61)	0.057	0.910	54 (45–62)	56 (47–61)	0.033	0.459
Sex			0.116	0.379			0.078	0.601
Male	99 (90.8)	109 (87.2)			82 (90.1)	84 (92.3)		
Female	10 (9.2)	16 (12.8)			9 (9.9)	7 (7.7)		
ECOG PS			0.153	0.245			0.067	0.649
0	95 (87.2)	102 (81.6)			79 (86.8)	81 (89.0)		
1	14 (12.8)	23 (18.4)			12 (13.2)	10 (11.0)		
HBsAg			0.206	0.115			<0.001	>0.999
Negative	18 (16.5)	12 (9.6)			11 (12.1)	11 (12.1)		
Positive	91 (83.5)	113 (90.4)			80 (87.9)	80 (87.9)		
Child–Pugh class			0.013	0.920			<0.001	>0.999
A	99 (90.8)	114 (91.2)			84 (92.3)	84 (92.3)		
B7	10 (9.2)	11 (8.8)			7 (7.7)	7 (7.7)		
BCLC stage			0.198	0.131			<0.001	>0.999
B	31 (28.4)	25 (20.0)			24 (26.4)	24 (26.4)		
C	78 (71.6)	100 (80.0)			67 (73.6)	67 (73.6)		
α-Fetoprotein			0.153	0.244			<0.001	>0.999
≤400 ng/mL	68 (62.4)	87 (69.6)			58 (63.7)	58 (63.7)		
>400 ng/mL	41 (37.6)	38 (30.4)			33 (36.3)	33 (36.3)		
Tumor size (cm)	7.9 (5.5–11.0)	9.1 (6.9–12.2)	0.234	0.116	8.9 (5.5–11.4)	9.1 (6.7–11.8)	0.097	0.882
Tumor number			0.061	0.644			0.044	0.766
≤3	56 (51.4)	68 (54.4)			48 (52.7)	50 (54.9)		
>3	53 (48.6)	57 (45.6)			43 (47.3)	41 (45.1)		
PVTT			0.178	0.175			0.044	0.767
Absent	55 (50.5)	52 (41.6)			44 (48.4)	42 (46.2)		
Present	54 (49.5)	73 (58.4)			47 (51.6)	49 (53.8)		
Extrahepatic spread			0.094	0.473			0.090	0.542
Absent	63 (57.8)	78 (62.4)			54 (59.3)	58 (63.7)		
Present	46 (42.2)	47 (37.6)			37 (40.7)	33 (36.3)		
Prior TACE			0.076	0.562			0.048	0.748
No	72 (66.1)	87 (69.6)			62 (68.1)	64 (70.3)		
Yes	37 (33.9)	38 (30.4)			29 (31.9)	27 (29.7)		
PD-(L)1 inhibitor			0.086	0.806			0.079	0.868
Sintilimab	53 (48.6)	65 (52.0)			46 (50.5)	48 (52.7)		
Tislelizumab	22 (20.2)	25 (20.0)			22 (24.2)	19 (20.9)		
Others	34 (31.2)	35 (28.0)			23 (25.3)	24 (26.4)		

Data are median (interquartile range) or n (%)

BCLC, Barcelona Clinic Liver Cancer; ECOG PS, Eastern Cooperative Oncology Group performance status; HBsAg, hepatitis B surface antigen; PD-(L)1, programmed cell death–(ligand)1; PSM, propensity score matching; PVTT, portal vein tumor thrombus; SIRT-L-P, selective internal radiation therapy combined with lenvatinib and PD-(L)1 inhibitor; SMD, standardized mean difference; TACE, transarterial chemoembolization; TACE-L-P, transarterial chemoembolization combined with lenvatinib and PD-(L)1 inhibitor.

^*^ Mann–Whitney *U* for continuous variables and Chi-squared test or Fisher exact test for categorical variables were applied.

In the matched cohort, the median age was 54 (interquartile range [IQR]: 45–62) years, and 82 patients (90.1%) were male in the SIRT-L-P group, while the median age was 56 years (IQR: 47–61), and 84 patients (92.3%) were male in the TACE-L-P group. The median tumor size was 8.9 cm (IQR: 5.5–11.4) in the SIRT-L-P group and 9.1 cm (IQR: 6.7–11.8) in the TACE-L-P group. The majority of patients had advanced disease in both groups, with 73.6% classified as BCLC stage C. Additionally, about 30% patients had undergone prior TACE.

### Efficacy outcomes

In the matched cohort, the median follow-up period was 12.2 months (IQR, 6.9–20.4) for SIRT-L-P group and 11.4 months (IQR, 7.7–17.5) for TACE-L-P group (*P* = 0.657). In the SIRT-L-P group, 80 patients underwent one SIRT procedure, and 11 patients underwent two SIRT procedures. The median administered activity of ^90^Y was 1.84 GBq (IQR: 1.38–2.95), with the median absorbed dose to tumors of 150 Gy (IQR: 120–175) and the median estimated dose to the lungs of 4.9 Gy (IQR: 2.0–10.6). Patients in the TACE-L-P group underwent a total of 296 TACE procedures, with a mean of 3.3 ± 1.4. The median number of target vessels at the first on-study SIRT/TACE session was 2 (IQR, 1–3; range 1–5) in both groups, with no significant difference between groups (Mann–Whitney *U* = 4496.0, *Z* = 1.045, *P* = 0.296; Supplemental Digital Content Figure S2, available at:http://links.lww.com/JS9/G421). The median duration of lenvatinib administration was 12.4 months (IQR, 7.8–19.3) in the SIRT-L-P group and 8.8 months (IQR, 4.9–15.3) in the TACE-L-P group. The median duration of PD-(L)1 inhibitor administration was 15.7 months (IQR, 10.8–19.5) in the SIRT-L-P group and 9.6 months (IQR, 5.5–16.1) in the TACE-L-P group. During follow-up, 16 patients (17.6%) in the SIRT-L-P group underwent surgical interventions when there was shrinkage of tumor (12 liver resections and 4 transplantations), compared to 5 (5.5%) in the TACE-L-P group (4 liver resections and 1 transplantation) (*P* = 0.011).

**Figure 2.**
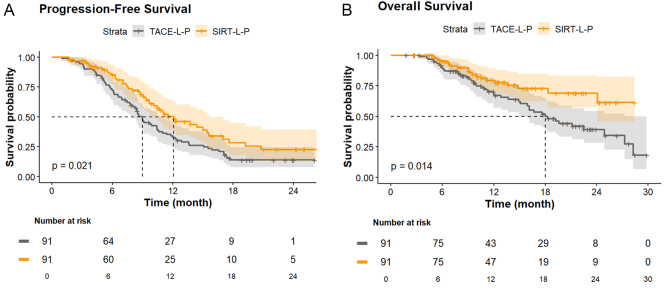
Kaplan–Meier analysis of progression-free survival (A) and overall survival (B). SIRT-L-P, selective internal radiation therapy combined with lenvatinib and PD-(L)1 inhibitor; TACE-L-P, transarterial chemoembolization combined with lenvatinib and PD-(L)1 inhibitor; PD-(L)1, programmed cell death–(ligand)1.

**Figure 3.**
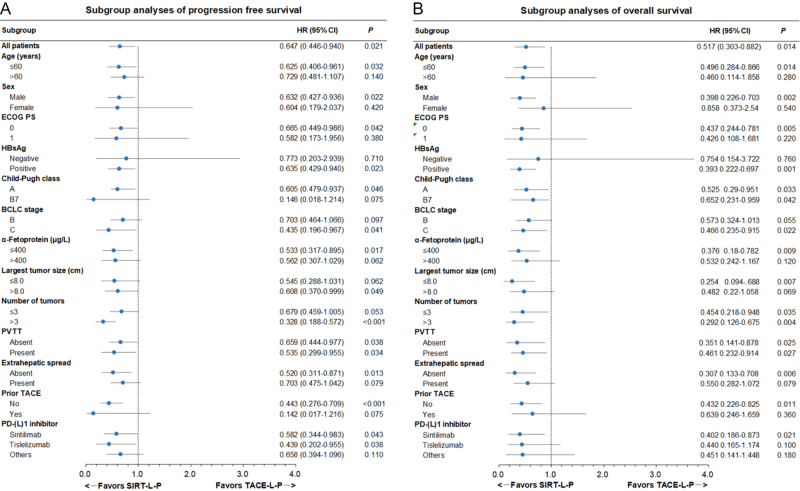
Subgroup analysis of progression-free survival (A) and overall survival (B). HR, hazard ratio; CI, confidence interval; ECOG PS, Eastern Cooperative Oncology Group performance status; HBsAg, hepatitis B surface antigen; BCLC, Barcelona Clinic Liver Cancer; PVTT, portal vein tumor thrombus; TACE, transarterial chemoembolization; PD-(L)1, programmed cell death–(ligand)1.

During follow-up, 45 (49.5%) patients in the SIRT-L-P group and 72 (79.1%) patients in the TACE-L-P group had tumor progression or death. Subsequent treatments initiated after tumor progression or intolerance to the initial therapy did not differ significantly between groups (all *P* > 0.05; Supplemental Digital Content Table S1, available at:http://links.lww.com/JS9/G421). Median PFS was 12.1 months (95% CI, 10.1–15.6) in the SIRT-L-P group, which was significantly longer than 9.0 months (95% CI, 8.0–10.6) in the TACE-L-P group (hazard ratio [HR] 0.647 [95% CI, 0.446–0.940], *P* = 0.021) (shown in Figure 2A). When surgery events were not censored in PFS analysis, the median PFS was 13.4 months (95% CI, 10.3–16.3) in the SIRT-L-P group versus 9.6 months (95% CI, 7.8–10.2) in the TACE-L-P group (HR 0.610 [95% CI, 0.411–0.853], *P* = 0.010). The treatment effect in PFS remained consistent in direction and magnitude, supporting the robustness of the primary findings. The median OS in SIRT-L-P group was not reached (95% CI, 24.1 months–NE) with the 1-, and 2-year OS rate of 79.3%, and 68.9%, respectively, while the median OS in TACE-L-P group were 18.0 months (95% CI, 15.1–28.2) with the 1-, and 2-year OS rate were 68.6% and 39.1%, respectively (HR 0.517 [95% CI, 0.303–0.882], *P* = 0.014) (shown in Figure 2B). Multivariate analyses identified that treatment with SIRT-L-P was an independent protective factor for PFS (HR 0.566 [95% CI, 0. 386-0. 830], *P* = 0.004) and OS (HR 0. 429 [95% CI, 0.250–0.737], *P* = 0.002) (Table 2). Subgroup analyses showed that consistent trends of lower risk of disease progression and death were achieved with SIRT-L-P over TACE-L-P in all the subgroups (shown in Figure 3). There was no statistically significant interaction between treatment method and any covariates in predicting PFS and OS (all *P*_interaction_ > 0.1). The ORR for the SIRT-L-P group was 78.0% (95% CI, 69.4–86.7%) according to mRECIST, compared to 61.5% (95% CI, 51.4–71.7%) for the TACE-L-P group (OR 2.219 [95% CI, 1.156–4.257], *P* = 0.017). Per RECIST 1.1, the ORR was 56.0% (95% CI, 45.7-66.4%) for the SIRT-L-P group and 35.2% (95% CI, 25.2-45.2%) for the TACE-L-P group (OR 2.351 [95% CI, 1.294–4.271], *P* = 0.005). Sensitivity analyses with three different approaches (PSM with caliper = 0.05, optimal matching method, and IPTW), together with a sensitivity analysis restricted to patients without prior TACE in which PSM was re-performed (PFS HR 0.401 [95% CI, 0.245–0.655]; OS HR 0.431 [95% CI, 0.220–0.845]; Supplemental Digital Content Figure S3, available at:http://links.lww.com/JS9/G421 and Supplemental Digital Content Figure S4, available at:http://links.lww.com/JS9/G421), confirmed the above findings and demonstrated the robustness of the primary PSM analysis (detailed in Supplementary Materials).

**Table 2:** Analyses of prognostic factors for progression-free survival and overall survival in the matched cohorts.

Factor	Univariate analysis		Multivariate analysis	
	HR (95% CI)	*P*	HR (95% CI)	*P*
Analyses for PFS				
Treatment (SIRT-L-P)	0.647 (0.446–0.940)	0.021	0.566 (0.386–0.830)	0.004
Age (years)	0.990 (0.974–1.005)	0.199		
Sex (male)	1.385 (0.723–2.654)	0.327		
HBsAg (positive)	1.089 (0.610–1.945)	0.772		
ECOG PS (1)	1.875 (1.045–3.365)	0.035	1.870 (1.036–3.375)	0.038
Child–Pugh class (B)	1.444 (0.729–2.859)	0.292		
BCLC stage (C)	2.098 (1.333–3.300)	0.001	—	0.736
α-Fetoprotein (≥ 400 μg/L)	1.189 (0.817–1.729)	0.366		
Largest tumor size (cm)	1.009 (0.964–1.056)	0.703		
Number of tumors (>3)	1.620 (1.115–2.355)	0.011	1.774 (1.206–2.610)	0.004
PVTT (present)	1.482 (1.026–2.141)	0.036	1.562 (1.077–2.266)	0.019
Extrahepatic spread (present)	1.970 (1.363–2.847)	<0.001	1.988 (1.374–2.876)	<0.001
Prior TACE (yes)	1.328 (1.074–2.265)	0.027	—	0.319
PD-(L)1 inhibitor (Tislelizumab^a^)	0.883 (0.561–1.389)	0.590		
PD-(L)1 inhibitor (Others^a^)	1.357 (0.869–2.120)	0.180		
Analyses for OS				
Treatment (SIRT-L-P)	0.517 (0.303–0.882)	0.014	0.429 (0.250-0.737)	0.002
Age (years)	1.007 (0.986–1.028)	0.539		
Sex (male)	2.177 (0.788–6.014)	0.133		
HBsAg (positive)	1.253(0.539–2.912)	0.600		
ECOG PS (1)	2.411 (1.206–4.822)	0.013	—	0.612
Child-Pugh class (B)	3.411 (1.760–6.609)	<0.001	3.893 (1.970–7.693)	< 0.001
BCLC stage (C)	2.150 (1.119–4.132)	0.022	—	0.683
α-Fetoprotein (≥ 400 μg/L)	1.465 (0.883–2.430)	0.140		
Largest tumor size (cm)	1.011 (0.951–1.074)	0.734		
Number of tumors (> 3)	1.007 (0.610–1.661)	0.979		
PVTT (present)	1.484 (0.992–2.455)	0.053	1.626 (0.972–2.722)	0.064
Extrahepatic spread (present)	1.765 (1.074–2.900)	0.025	1.902 (1.153–3.137)	0.012
Prior TACE (yes)	1.098 (0.679–1.862)	0.821		
PD-(L)1 inhibitor (Tislelizumab^*^)	0.867 (0.461–1.630)	0.658		
PD-(L)1 inhibitor (Others^*^)	1.305 (0.723–2.355)	0.377		

Analyses were performed using Cox proportional hazard regression model.

CI, confidence interval; ECOG PS, Eastern Cooperative Oncology Group performance status; HBsAg, hepatitis B surface antigen; HR, hazard ratio; OS, overall survival; PFS, progression-free survival; PD-(L)1, programmed cell death–(ligand)1; PVTT, portal vein tumor thrombus; SIRT-L-P, selective internal radiation therapy combined with lenvatinib and PD-(L)1 inhibitor; TACE, transarterial chemoembolization.

^a^ Compared to sintilimab

### Safety

Treatment-related AEs are shown in Table 3. No grade 4/5 AE occurred. Compared to the TACE-L-P group, there is a statistically significantly lower overall incidence of AEs of any grade in the SIRT-L-P group (83.5% vs 95.6%, *P* = 0.008). Notably, lower incidences of postembolization syndrome and liver toxicity in the SIRT-L-P group were reported: fever (3.3% vs 53.8%), abdominal pain (13.2% vs 65.9%), nausea/vomiting (3.3% vs 16.5%); and ALT evaluation (15.4% vs 25.3%) and AST evaluation (15.4% vs 31.9%). Grade 3 abdominal pain occurred exclusively in TACE-L-P group (11.0%). The incidences of grade 3 AEs were not significantly different between the groups (24.2% vs 30.8%, *P* = 0.319). The most common grade 3 AE in both groups was hypertension (14.3% and 15.4%) related to lenvatinib. Dose interruption and/or reduction due to AE happened in 18.7% and 20.9% of patients, respectively, in the SIRT-L-P group and the TACE-L-P group, and dose discontinuation in the 8.8% and 6.6% patients.

**Table 3:** Adverse events in the two matched groups.

Adverse events	Any Grade			Grade 3		
	SIRT-L-P (*N* = 91)	TACE-L-P (*N* = 91)	*P* value a	SIRT-L-P (*N* = 91)	TACE-L-P (*N* = 91)	*P-*value a
Overall incidence	76 (83.5)	87 (95.6)	0.008	22 (24.2)	28 (30.8)	0.319
Post-embolization syndrome						
Fever	3 (3.3)	49 (53.8)		0	0	
Abdominal pain	12 (13.2)	60 (65.9)		0	10 (11.0)	
Nausea/vomiting	3 (3.3)	15 (16.5)		0	0	
Liver dysfunction						
Hypoalbuminemia	8 (8.8)	14 (15.4)		0	3 (3.3)	
Blood bilirubin increase	10 (11.0)	14 (15.4)		0	5 (5.5)	
ALT elevation	18 (15.4)	23 (25.3)		3 (3.3)	6 (6.6)	
AST elevation	18 (15.4)	29 (31.9)		0	4 (4.4)	
Hematologic toxicity						
Anemia	9 (9.9)	11 (12.1)		2 (2.2)	3 (3.3)	
Leucocyte decrease	18 (15.4)	9 (9.9)		3 (3.3)	1 (1.1)	
Neutrophil decrease	14 (15.4)	9 (9.9)		3 (3.3)	5 (5.5)	
Platelet decrease	29 (31.9)	15 (16.5)		3 (3.3)	4 (4.4)	
Diarrhea	15 (16.5)	11 (12.1)		0	0	
Fatigue	24 (26.4)	16 (17.6)		1 (1.1)	0	
Segmental bile duct dilatation	4 (4.4)	7 (7.7)		0	0	
Cholecystitis	4 (4.4)	4 (4.4)		0	0	
Ascites/ Pleural effusion	5 (5.5)	7 (7.7)		0	0	
Gastrointestinal hemorrhage	2 (2.2)	4 (4.4)		2 (2.2)	4 (4.4)	
Inguinal hematoma	3 (3.3)	1 (1.1)		0	0	
Hypertension	29 (31.9)	30 (33.0)		13 (14.3)	14 (15.4)	
Proteinuria	15 (16.5)	13 (14.3)		0	0	
Hand-foot syndrome	23 (25.3)	20 (22.0)		0	0	
Pneumonitis	3 (3.3)	1 (1.1)				
Gastric ulcer	3 (3.3)	2 (2.2)		0	0	
Weight loss	26 (28.6)	26 (28.6)		2 (2.2)	2 (2.2)	
Decreased appetite	18 (19.8)	17 (18.7)		2 (2.2)	1 (1.1)	
Hypothyroidism	19 (20.9)	17 (18.7)		0	0	
Pruritus	14 (15.4)	17 (18.7)		0	0	
Rash	17 (18.7)	14 (15.4)		0	0	
Arthralgia	10 (11.0)	9 (9.9)		0	0	
Gingival bleeding	8 (8.8)	10 (11.0)		0	0	
Edema	7 (7.7)	9 (9.9)		0	0	
Infusion reaction	3 (3.3)	2 (2.2)		0	0	
Hyperglycemia	2 (2.2)	4 (4.4)		0	0	

Data are *n* (%). No grade 4/5 adverse events were recorded.

AST, aspartate aminotransferase; ALT, alanine aminotransferase; SIRT-L-P, selective internal radiation therapy combined with lenvatinib and PD-(L)1 inhibitor; TACE, transarterial chemoembolization combined with lenvatinib and PD-(L)1 inhibitor.

^a^ Chi-squared test was performed; specific AE category comparisons were omitted to avoid inflation of type I error due to multiple testing.

### Exploratory study of immune biomarker-based risk stratification after SIRT

At 3–4 weeks after SIRT, systemic immune modulation manifested as significant reductions in circulating neutrophils (median Δ = − 0.99 × 10^9^/L) and lymphocytes (median Δ = − 0.45 × 10^9^/L), and increase in neutrophil-to-lymphocyte ratio (NLR; median 4.72 vs 3.55) and peripheral CD8 + T-cell proportion was observed (median Δ = + 4.2%; all *P* < 0.05) (shown in Supplemental Digital Content Table S2, available at:http://links.lww.com/JS9/G421; Supplemental Digital Content Figure S5, available at:http://links.lww.com/JS9/G421). Additionally, the post-SIRT NLR had nearly no association with baseline NLR (Spearman’s *ρ* = 0.038, *P* = 0.784). Among these variables, high post-SIRT NLR predicted inferior PFS (HR 2.55 [95% CI, 1.05–6.21], *P* = 0.040), whereas high post-SIRT CD8 + T-cell proportion showed a protective trend for PFS (HR 0.85 [95% CI 0.71–1.02], *P* = 0.08). However, none of the parameters demonstrated a significant predictive value for OS (shown in Supplemental Digital Content Figure S6, available at: http://links.lww.com/JS9/G421).

A significant interaction between NLR and CD8 + T-cells was observed in predicting PFS (*P*_interaction_ = 0.015) and OS (*P*_interaction_ = 0.037), suggesting the potential for a composite NLR-CD8 biomarker. Stratification by post-SIRT NLR and CD8 identified three prognostic tiers: low risk (NLR <4.72 + CD8 > 27.2%), intermediate-risk (NLR <4.72 + CD8 ≤ 27.2% or NLR ≥4.72 + CD8 > 27.2%), and high-risk (NLR ≥4.72 + CD8 ≤ 27.2%). PFS risk increased incrementally across risk tiers (low→intermediate→high) (HR 2.74, 95% CI 1.15–6.50; *P*_trend_ = 0.016 by ordinal Cox analysis) (shown in Supplemental Digital Content Figure S7A, available at: http://links.lww.com/JS9/G421). Furthermore, NLR-CD8 tier retained independent prognostic significance for PFS in multivariable models adjusting for confounding factors (HR 2.32 per tier [95% CI, 1.15–4.66], *P*_trend_ = 0.018), whereas individual parameters of NLR and CD8 + T-cell proportion lost predictive value when adjusted for confounders. In addition, high-risk tier exhibited markedly worse OS (HR 4.91 [95% CI, 2.54–7.63], *P* = 0.007) versus low/intermediate-risk tiers (shown in Supplemental Digital Content Figure S7B, available at: http://links.lww.com/JS9/G421), and retained independent prognostic significance for OS in multivariable models (HR 2.85 [95% CI, 1.22–6.66] *P* = 0.015) (Supplemental Digital Content Table S3, available at: http://links.lww.com/JS9/G421).

## Discussion

To the best of our knowledge, this study represents the first comprehensive analysis of SIRT in a Chinese HCC population. Our results demonstrated that SIRT-L-P significantly improved PFS, OS, and ORR compared to TACE-L-P in Chinese patients with high-tumor burden HCC, i.e., beyond the up-to-seven criteria or with PVTT. The robustness of these findings is supported by sensitivity analyses confirming matching process validity, a sensitivity analysis restricted to patients without prior TACE, and subgroup analyses revealing consistent therapeutic benefits across diverse patient populations, including PVTT-present patients (HR 0.535, 95% CI 0.299–0.955), reinforcing broad clinical applicability of SIRT-L-P in advanced disease phenotypes.

The TACE-L-P cohort in our study achieved median PFS and OS of 9.0 months and 18.0 months, respectively, aligning with outcomes from multiple studies^[4,5,22]^, including nationwide scale studies CHANCE001 and CHANCE2201^[4,5]^, which investigated combination of TACE with molecular-targeted therapy and PD-(L)1 inhibitors in patients with intermediate-advanced HCC (median PFS 7.0–9.9 months; OS 16.9–22.6 months). These results confirm TACE-L-P as a viable treatment option within the context of similar therapies, and suggests that our data are representative of real-world outcomes. Notably, recent pivotal phase 3 trials (LEAP-012, EMERALD-1) have confirmed the viability of combining TACE with targeted therapy and PD-(L)1 inhibitors in unresectable HCC ranging from early to advanced stage^[6,7]^, establishing a conceptual framework that provided the basis for our investigation of SIRT-based combination therapy.

Two factors likely underlie the superior efficacy of SIRT-L-P over TACE-L-P in high-tumor-burden HCC: stronger local control from direct radiotherapeutic effect and more favorable immunologic synergy. TACE, relying on embolization-induced ischemia, can be limited by residual perfusion from the portal venous supply and rapid revascularization leading to incomplete embolization^[23]^. Besides, TACE carries high risks of postembolization syndrome and suboptimal responses in large tumors or those with macrovascular invasion (e.g., PVTT)^[11,12]^. In contrast, SIRT delivers territorial radiation with minimal embolic effect and does not depend on arterial stasis. In HCC patients who are poor candidates for TACE, such as those with high-tumor burden, PVTT, or prior TACE failure, SIRT has shown superior local tumor control versus TACE, with longer time to progression (HR 0.54–0.61) and lower local recurrence (18% vs 51.7%) in meta-analyses^[24]^. This advantage of SIRT in local efficacy likely lays the foundation for improved outcomes when combined with systemic therapy in our high-tumor burden cohort.

Considering the immunologic synergy, while some studies suggested that TACE may induce weak or functionally limited antitumor responses insufficient to prevent relapse, as seen in impaired IFN-γ production and exhaustion markers in tumor-associated antigen-specific CD8 + T cells^[25]^, it was also reported to foster an immunosuppressive tumor microenvironment derived from embolization-driven ischemia, characterized by reduced CD8 + T-cell infiltration and elevated immunosuppressive macrophages^[14,15]^. In contrast, SIRT elicits anti-tumor immune activation locally and systemically. SIRT induces radiation-mediated tumor cell death, potentially enhancing antigen presentation and creating an “in situ vaccine” effect^[10]^. Emerging evidence from human HCC cohorts indicates that SIRT–treated tumors show enriched granzyme B^+^ tumor-infiltrating lymphocytes (CTLs) with increased CD8^+^ T cells, CD56^+^ NK, and CD8^+^CD56^+^ NKT-like cells and up-regulated innate/adaptive activation programs, with chemokine signaling (CCL5–CCR5, CXCL16–CXCR6) supporting recruitment of granzyme B^+^ CD8^+^ T cells to tumors, while in peripheral blood the frequencies of TNF-α^+^ CD4^+^ and TNF-α^+^ CD8^+^ T cells increased, together with a higher proportion of antigen-presenting cells^[13,26]^. This SIRT–induced immunomodulation peaks at about 1 month and lasts for nearly 3–6 months, providing a clinically actionable window for initiating combination immunotherapy. In head-to-head comparison with TACE, SIRT significantly elevates tumor-infiltrating lymphocytes (CD4 + and CD8 + subsets) and granzyme B expression in a radiation dose-dependent manner, highlighting its unique immunogenic potential which is not shown in TACE^[10]^. This mechanistic divergence in anticancer immune likely underlies the significant better outcomes in SIRT-L-P group.

When combined with PD-(L)1 inhibitors alone, previous studies report median PFS of 5.9–9.95 months^[27–29]^ – lower than the 12.1 months observed in our SIRT-L-P cohort, even though patients in our study had predominantly advanced disease and high-tumor burden. This disparity highlights lenvatinib’s critical role in the combination: its dual inhibition of VEGFR/FGFR pathways reduces pathological angiogenesis while promoting vascular normalization, thereby improving tumor oxygenation and radiosensitivity^[16–18]^. Lenvatinib was reported to show clinical benefits in advanced HCC with high-tumor burden^[30]^ and have better efficacy than sorafenib when combined with radiotherapy in HCC with PVTT^[16]^. Concurrently, lenvatinib modulates the tumor immune microenvironment by decreasing immunosuppressive tumor-associated macrophages and enhancing cytotoxic T-cell activity, creating synergistic effects with PD-(L)1 blockade^[31]^. This multidimensional approach appears to overcome resistance mechanisms more effectively than dual-component therapies.

Our findings revealed a paradoxical immune dynamic following SIRT, characterized by concurrent systemic inflammation (evidenced by NLR reset) and a significant increase in CD8 + T-cell proportion. This duality aligns with prior observations that SIRT simultaneously enhances tumor immunogenicity (CD8 + T-cell activation)^[10,13]^ and induces lymphocyte deletion^[32]^. Besides, CD8 + T-cell proportion increase may also be due to the survival and fast recovery of memory CD8 + T-cell subsets after radiation exposure^[33]^. Notably, our study found the interaction on efficacy predicting between elevated NLR and CD8 + T-cell proportion, suggesting that systemic inflammation might paradoxically attenuate antitumor immunity despite peripheral CD8 + increase. This may be elucidated by studies showing negative correlations between NLR and tumor-infiltrating CD8 + T cells^[34]^. This interaction prompted our NLR-CD8 composite risk model, which outperformed the individual biomarkers in predicting PFS and OS. Future prospective studies are warranted to refine and validate this model for early identification of SIRT-treated patients who may benefit from adjuvant immunotherapy, and further elucidate the functional phenotypes of CD8 + T-cell subsets after SIRT.

The safety profile of SIRT-L-P demonstrated meaningful advantages over TACE-L-P in reducing treatment-related AEs (83.5% vs 95.6%, *P* = 0.008), particularly post-embolization syndrome (fever, abdominal pain, and nausea/vomiting) and liver toxicity (ALT and AST elevation). This aligns with the inherent differences between SIRT and TACE mechanisms, where SIRT minimizes acute inflammatory responses to ischemia resulting from vessel embolism compared to TACE. Radiation-specific toxicities remained uncommon (no radiation-induced liver disease cases or grade ≥3 gastrointestinal or respiratory toxicity) – outcomes attributable to a strict and standardized pre-SIRT mapping procedure, meticulous dosimetry and (super-)selective microsphere targeting. Incidences of grade 3 AEs were comparable (24.2% vs 30.8%, *P* = 0.319), with lenvatinib-related hypertension being the most frequent in both groups (14.3% vs 15.4%). Dose adjustments and discontinuations were similar (18.7% vs 20.9%; 8.8% vs 6.6%), underscoring consistent systemic therapy tolerability. These findings position SIRT-L-P as a safer locoregional option for HCC patients vulnerable to embolization sequelae, and emphasize the need for vigilant blood pressure monitoring during lenvatinib use. Regarding the safety of interventional radiologists performing intra-arterial infusion of ^90^Y microspheres, published dosimetry shows very low occupational radiation exposure during SIRT when standard shielding is used^[35]^; our centers follow routine personal monitoring programs, and exposure doses lie well within regulatory limits.

This study has several limitations. The retrospective nature may introduce the potential selection bias, as indicated by the discrepancies in baseline characteristics before matching. To mitigate this, we rigorously performed PSM with sensitivity analyses confirming robustness of study results. Second, various kinds of PD-(L)1 inhibitors were utilized. Notably, all agents complied with international or Chinese HCC management guidelines, and were well balanced between the two groups. By pooling agents with shared targets and mechanisms, we aimed to validate a class-effect-based therapeutic strategy – an approach successfully implemented in other real-world studies^[9,10]^. Third, patient-level socioeconomic status was not captured, precluding direct adjustment; since SIRT has higher per-session charges, residual confounding by economic capacity cannot be completely excluded. However, aligned follow-up schedule, different session frequency, and comparable post-progression subsequent treatments between groups likely attenuate real-world differences. Finally, tumor infiltrating lymphocytes and functional phenotypes of CD8 + T-cell subsets were not tested because of the retrospective design, and an optimal cut-off value was not determined by Receiver Operating Characteristic analysis because of a limited sample size. However, this is only an exploratory study for post-SIRT immunologic model, and further studies are warranted to validate the model.

In conclusion, this study provides the first clinical evidence indicating that SIRT-L-P significantly improves efficacy endpoints, including PFS, OS, and ORR, compared to TACE-L-P, while maintaining superior tolerability in Chinese patients with high-tumor burden HCC. Moreover, post-SIRT NLR-CD8 tiers may serve as a predictive biomarker for early identification of patients who are most likely to benefit from this combination therapy. These findings highlight the potential of SIRT-L-P as a viable therapeutic strategy for unresectable HCC. However, prospective trials with mechanistic investigations are warranted to validate these results and optimize patient selection criteria.

## Data Availability

The datasets used or analyzed during this study are available from the corresponding author upon reasonable request.

## Research registration unique identifying number (UIN)

This study is registered at ClinicalTrials.gov, ClinicalTrials. gov ID is NCT06916546; the registration center is: The Second Affiliated Hospital of Guangzhou Medical University; the hyperlink to the registration is: https://clinicaltrials.gov/study/NCT06916546.

## Assistance with the study

The authors thank Peizhen Zhao (Dermatology Hospital of Southern Medical University, Guangzhou, China) who specializes in biostatistics for providing statistical advice.
